# Mr. Lodge's Observations on Suffocation

**Published:** 1805-08-01

**Authors:** Lodge Hall

**Affiliations:** Licentiate of the Royal College of Surgeons, in Ireland. (Medical Staff.) Cork


### To the Editors of the Medical and Physical Journal.

GENTLEMEN,

THE following case appearing to me and others, who were witnesses of it, to be one which has not before been taken notice of; you will oblige me by giving it a place in your useful Miscellany, that the public may be aware of the possibility of it, and by their united wisdom devise means of prolonging to society the lives of perhaps some useful individuals.

J. M. aged 22 years, a private soldier, was admitted into the General Hospital here on the 4th of July, labouring under Cynanche Tonsillaris; he had been four days ill and had taken no medicine whatever, not having been reported sooner.

His skin was at this time hot, his pulse pretty quick, and rather small, and he was costive; he breathed with some difficulty, though the tonsils and uvula were inconsiderably swelled. He appeared as if frightened, and said he never remembered being ill before, which seemed to account for it.

A saline laxative medicine was administered, and a blister was applied to the external fauces. Being visited in the evening, he said he was much better, but spoke in a hurried manner, and seemingly in terror, as in the morning; his breathing was not relieved, though he persisted in saying he was much better; the medicine had operated several times. On inspecting the throat, the tonsils and uvula were not observably altered. His skin still continuing hot and his pulse quick, he was ordered a diaphoretic mixture, composed of aq. ammon. acetat. & vin. antim. The morning following (5th of July) he was much worse. His countenance had a remarkable dejected appearance; I think it might be compared to the countenance often observable on the accession of a bad case of typhus fever. His pulse was small and labouring, his throat externally somewhat tumefied, occasioned probably by the blister; his inspiration was performed with the greatest difficulty,* the expiration almost imperceptibly, it was so very quick; and both together produced a remarkable sound,

* But I observed he did not attempt to assist it by putting his hands on his head or laying hold of any part of the bedstead, as asthmatics are sometimes observed to do.

sound, which induced the belief that the trachea might be the seat of the disease. On attempting to swallow any fluid, part of it was immediately ejected through the nose, and most part of the remainder after a short time; it is even probable *none* reached the stomach. Inspecting the throat internally, the uvula and tonsils were certainly larger, but in the opinion of several medical gentlemen present, by no means so much as to account for the very great difficulty of breathing observable; on the uvula were perceived some little asperities, as if it had burst and discharged some fluid. Bronchotomy, or (speaking more correctly) tracheotomy, was now thought of, but rejected upon the supposition that the trachea was affected perhaps to the lungs. The uvula and tonsils were pierced, but without any thing being seen to discharge; he was directed to inhale the steam from warm water with some vinegar, and a large blister was applied to his neck posteriorly; he had been rather purged since the laxative was given the preceding day.

He died that evening apparently suffocated, it being the *sixth* day of his illness, and the *second* of his admission into the hospital.

On examination of the parts by dissection, the submaxillary glands appeared somewhat enlarged; one of the tonsils and the uvula were in a state of suppuration, but the pus too viscid to flow out; their size by no means great; the velum pendulum palati seemed thicker than natural; but the EPYGLOTTIS was considerably *enlarged, inflamed,* and in a *partial state of suppuration,* containing *viscid pus,* as before-mentioned, found in the uvula and one of the tonsils.

Every one present* agreed that this was sufficient to cause the difficulty of breathing and consequent death of the man. The parts contiguous were however examined, viz. the glottis, and all the parts contained within (or behind) the thyroid cartilage, and they were not diseased; the trachea was particularly noticed to be in a healthy state. The thorax being opened, the lungs appeared natural; but on pressing them, froth mixed with blood issued from the trachea; the consequence, of course, of suffocation.

If, contrary to my supposition, such cases as the above have been before related, I crave pardon of you, gentlemen, and of the public, for having obtruded this; I also beg excuse for its being related thus briefly, as I am not

 conscious

* Chiefly gentlemen of the medical staff.

conscious of having omitted any thing that might lead to the discovery of the disease in future. An ingenious person might liken the sound I have mentioned, as occasioned by the difficulty of breathing, to something familiar, but I really cannot think what to liken it to; however, I can safely aver, it was *not* like the ringing of a brazen tube; the similitude to which is mentioned by most authors as accompanying cynanche trachealis or the croup. The manner it was treated I by no means lay down as the best possible, were the disease known. I am sensible it may be deemed omissive; some may think bleeding ought to have been performed; but the pulse, during the *two days* he was in the hospital, did not authorize it, nor was the only inflammation visible at all alarming; and I must again repeat, that what caused the difficult respiration was not known: all I can say is, that with a strict adherence to truth, I have *simply* related the different particulars as they occurred; and all concerned in the treatment of the case will, I know, be glad if even by their errors being pointed out, others may be served. Many modern medical writers could, I make no doubt, with the above materials, form a small volume, and doom the unfortunate reader to be tossed through seas of irrelevant and extraneous matter before he should reach the much desired port of useful information; and often has it been the cause of much wonder in me, when perusing the works of one of these literary spiders, that the subject by being so unconscionably stretched through many pages, (perhaps some volumes) with various angles and contortions, has not at last been rent, and the unwary author assumed, instead thereof, *politics*, or some of the *fashionable topics of the day*. But lest I should myself err in like manner, I will here conclude; and am, &c.

*Cork, July* 10, 1805.

LODGE HALL,

Licentiate of the Royal College of Surgeons, in Ireland.

